# Effect of sodium bicarbonate and beta-alanine supplementation on maximal sprint swimming

**DOI:** 10.1186/1550-2783-10-52

**Published:** 2013-11-11

**Authors:** Antti A Mero, Petri Hirvonen, Janne Saarela, Juha J Hulmi, Jay R Hoffman, Jeffrey R Stout

**Affiliations:** 1 Department of Biology of Physical Activity, University of Jyväskylä, P.O. Box 35, Jyväskylä 40351, Finland; 2 Sport and Exercise Science, University of Central Florida, Orlando, Florida, USA

**Keywords:** Alkalosis, Anaerobic exercise, Buffer, Ergogenic aid

## Abstract

**Background:**

This study examined the effect of simultaneous supplementation of extracellular buffer sodium bicarbonate (SB) and intracellular buffer beta-alanine (BA) on maximal sprint swimming.

**Methods:**

Thirteen competitive male swimmers completed 4 different treatments (placebo [PL], SB, BA + PL, and BA + SB) in a crossover procedure. PL or SB supplementation (0.3 g/kg body weight) was ingested 60 min before two maximal 100-m freestyle swims that were performed with a passive recovery of 12-min between each swim. Because of the known long washout period for carnosine, four weeks of BA supplementation (4.8 g per day) was started after the first week of PL or SB supplementation and performance testing.

**Results:**

The first maximal swims were similar, but the increase in time of the second versus the first 100-m swimming time was 1.5 s more (p < 0.05) in PL than in SB. Blood pH values were significantly (p < 0.05) greater in the SB and in the BA + SB groups compared to the PL and BA + PL values. There were no differences in peak blood lactate between the treatments.

**Conclusion:**

Supplementing with SB prior to performing maximal sprint swimming with repetitions under 60 s improves performance. However, co-supplementation with SB and BA did not confer any added benefit on maximal swim performance.

## Background

During intensive anaerobic exercise with a large glycolytic component, one major cause of fatigue is believed to be acidosis caused by high levels of hydrogen ions (H^+^) in the muscle fibers. The increase in (H^+^) corresponds to a decrease in muscle and blood pH [1], can slow glycolysis [2], interfere with calcium release from the endoplasmic reticulum and calcium ion binding [3,4], and increase the perception of fatigue after some types of exercises [5]. A number of buffers can be used by the body, but the primary method for buffering the H^+^ is thought to be either bicarbonate or hemoglobin [6]. For the past 35 years, several studies have investigated the use of sodium bicarbonate (SB) as an ergogenic aid. The participants have typically been men, and efficacy (improved performance and a decrease in H^+^ concentration after exercise) has generally been seen at doses of at least 0.3g· kg^-1^ body mass [7-9].

A recent meta-analysis by Carr et al. [10] suggests that ingestion of SB at 0.3 - 0.5g·kg^-1^ body mass improves mean power by 1.7 ± 2.0% during high-intensity races of short duration (1–10 min). Timing of ingestion ranging from 60 min - 180 min before exercise did not influence buffering capacity or the ergogenic potential of SB (0.3g·kg^-1^ body mass) as assessed by repeated sprint ability. However, visual analog scale scores indicated that at 180 minutes post-ingestion, an individual is less prone to experiencing significant gastrointestinal discomfort [11]. Gao et al. [3] and Siegler et al. [12] have demonstrated that swimmers ingesting 0.3g·kg^-1^ body mass of SB can enhance blood buffering potential and positively influence interval swim performance. Lindh and colleagues [13] have also shown that SB supplementation (0.3g·kg^-1^ body mass) can improve a single 200 m freestyle performance time in elite male competitors, most likely by increasing extra-cellular buffering capacity.

Beta-alanine (BA) is a non-essential amino acid that combines with L-histidine, to form the dipeptide carnosine. BA is thought to be the rate-limiting step in the synthesis of carnosine [14]. Carnosine acts as an intracellular buffer during high-intensity exercise [15-18], and elevations in muscle carnosine concentration have been demonstrated to enhance cycling capacity [18], ventilatory threshold, and delay fatigue [19]. A recent meta-analysis [20] has shown a significant ergogenic effect of BA supplementation during high intensity exercise lasting 60–240 s in duration. However, the efficacy of BA supplementation during single exercise durations shorter than 60 s durations is not clear. Although the efficacy of BA on repeated sprint performance is not very well known, studies examining BA and resistance training performance have indicated significant increases in training volume [21,22], suggesting that BA ingestion would be beneficial for repetitive high intensity exercise activities.

There appears to be only a limited number of studies that have examined a combination of two supplemental buffers on exercise performance. Mero and colleagues [23] indicated that the combined ingestion of SB and creatine (Cr) enhanced performance in two consecutive maximal effort 100-m swims with a 10 min recovery to a greater extent than ingestion of the supplements separately. Hoffman et al. [22] were the first to examine the combination of both BA and Cr supplements. Results of their study demonstrated that this combination significantly improved the training volume more than creatine alone. Specifically, improvements in training volume were found to be associated with significantly greater gains in lean body mass and decreases in fat mass. Sale et al. [24] investigated the effects of the combination of SB and BA (4 weeks loading) on high intensity cycling endurance performance and found that BA alone improved cycling capacity. Despite a 6 s improvement in time to exhaustion with the addition of SB, it did not reach statistical significance. In another cycling study [25] acute SB supplementation significantly improved 4-min cycling performance, but there seemed to be only a minimal additive effect of combined BA and SB supplementation. In the study by Hobson et al. [26] it was shown that both chronic BA and acute SB supplementation alone had positive effects on 2000 m rowing endurance performance. The addition of acute SB to chronic BA supplementation may further enhance rowing performance. Chronic BA and SB supplementation alone equally enhanced high-intensity intermittent maximal upper-body performance (4 × 30 s with three minutes recovery) in well-trained athletes and combining BA and SB promoted a clear additive ergogenic effect [27]. Ducker et al. [28] investigated if combining BA and SB could lead to enhanced repeated-sprint performance (3 sets; 6 × 20 m departing every 25 s, 4 minutes recovery between sets). They concluded that supplementation with acute SB improved repeated-sprint performance more than either a combination of SB and BA or BA alone. In a recent study [29] the swimmers swam maximally at first 200 m and then 100 m with 30 minutes recovery. BA and SB supplementation improved both 200 m and 100 m swimming performance. The co-ingestion of BA and SB induced a further nonsignificant improvement in performance. The performance time in 100 m was a little bit over 60 s (60–64 s). This time limit 60 s [20] is interesting in races e.g. in swimming (100 m) and in running (400 m). Earlier Sostaric et al. [30] reported that SB supplementation lowered circulating potassium, enhanced muscle potassium uptake and sodium delivery with alkalosis, but there are no studies with BA supplementation. These physiological changes are all interesting with preservation of membrane excitability during exercise [30]. Therefore, the purpose of present study was to examine more the effect of SB (extracellular buffer), BA (intracellular buffer) and the combination of SB with BA on a maximal sprint performance under 60 s in swimmers in a simulated competition.

## Methods

### Participants

Thirteen national and international level male swimmers (mean ± SD: age 20.5 ±1.4 years, body mass 80.1 ± 8.1 kg, height 188 ± 8 cm, haemoglobin 150 ± 6 g · l^-1^ (average of the first and third test day), 100 m freestyle record 54.44 ± 2.41 s) were recruited from the local swimming team to serve as participants. All swimmers exercised in the same training group. Each participant provided a written informed consent, and was free to withdraw from the study at any time. This study was approved by Ethics Committee of the local University.

### Experimental design and supplementation

**Figure 1:**
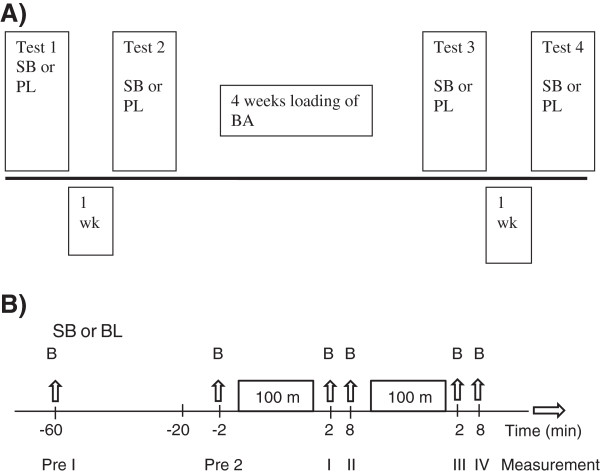
**Experimental design. A)** Swim test days 1–4, **B)** Timeline of each test day, SB = sodium bicarbonate, PL = placebo and BA = Beta-alanine supplementation, B = blood sample, 2 x 100 m swimming (swim 1 and 2).

Experimental design is shown in Figure 1. In the first part of the study the participants ingested gelatine covered capsules containing SB (1 g per capsule) or the placebo (calcium carbonate). Each participant was provided a dose equivalent to 0.3g·kg^-1^ body mass. The capsules were weighed to ensure the correct amount of substance in each capsule. Participants were provided with the SB supplement or with the placebo 60 minutes prior to performing the swimming protocol. This part of the experiments was randomized and double blinded. SB and calcium carbonate were acquired from the local pharmacy.

In addition to the acute SB or placebo ingestion, in the second part of the study the participants were provided a daily dose of BA for a 4-week period. Each participant was provided gelatine coated capsules, each containing 0.6 g of BA. Participants ingested eight capsules per day in 1.5 - 2 h intervals throughout the 4 week period; therefore the total consumption of BA per day was 4.8 g [31]. Participants were instructed to consume the capsules at the same time every day which was controlled verbally by the researchers. The subjects and the researchers knew that every subject was consuming BA during a 4-week period (unblinded). After the period the participants were provided with the SB supplement or with the placebo supplement 60 minutes prior to performing the swimming protocol. Also this part of the experiments was randomized and double blinded. The subjects were told to keep a food diary for 24 hours prior to all tests, and to maintain similar eating habits and food prior to each test. All tests occurred at the beginning of the week (Monday & Tuesday), following a rest day. BA for the current study was provided by Manninen Nutraceuticals Ltd. (Oulu, Finland). Beta-alanine was not tested for contamination with stimulants or anabolic agents, however, the sponsoring company had certification on the quality of their beta-alanine product that it did not contain any banned substances.

### Test day

The time line of the test day is shown in Figure 1B. On each test day participants consumed a self-prepared breakfast and arrived at the pool. Participants were grouped in pairs and were requested to arrive at 45 minute intervals to ensure a smooth flow of testing. Appointment times for each subject occurred at the same time of the day for both pre and post testing sessions. Actual performance testing took place in a 50-m pool during the afternoon. The water temperature during all tests was kept constant at 26.5° - 27.0°C and air temperature in the hall was 22° - 23°C. Participants provided their food diaries, and resting blood samples were obtained. They were then provided either with the SB supplement or the placebo 60 minutes prior to swimming. Following supplement ingestion the participants rested for 40 minutes by the pool. Easy walking and stretching was allowed during this time. After 40 minutes the subjects went to the pool to perform an 800-m standardized warm up. After the warm up, the actual test began. The test itself consisted of 2 × 100-m maximal freestyle sprints with a 12 min passive rest interval between each sprint. Just before the first swim, a blood sample was taken. The swimmers performed in pairs to create a competitive atmosphere and to motivate them to maximize their performance. The swimmers were paired according to their individual records in the 100-m freestyle. Every swim was timed with two experienced persons using stop-watches and their average value was used as the final swimming time. In all swimming times (n = 104) of the two timers, interclass correlation (ICC) was 0.99, standard error of measurement (SEM) was 0.16 seconds, and no significant difference was observed between the times of the two persons (57.2 ± 2.3 s and 57.2 ± 2.3 s). Subjects were also requested to report all any side effects to the investigators.

### Blood collection and analysis

On the test day a total of six blood samples were obtained from every subject at six measurement points (Figure 1B). Whole blood samples were taken from a finger by using a sterile lancet, the first drop of blood was discarded, and free flow blood was collected in a balanced heparin 200-mL blood gas capillary tube. Two pre-exercise samples were obtained at 60 min (Pre 1) and 2 min (Pre 2) prior to commencing exercise, and post exercise blood samples were taken 2- and 8-min following each swim (I – IV, respectively). In addition, on the first and third measurement day a blood sample (2 ml) was obtained from a forearm vein using a needle and syringe. Blood samples were collected into an EDTA-vacuum tube to analyse haemoglobin.

All blood samples were analysed within six hours after collection. Blood lactate (B-Lactate), blood pH (B-pH), blood potassium (B-Potassium), blood sodium (B-Sodium), blood bicarbonate (B-Bicarbonate), blood base excess (B-Base excess) were analysed from all samples. The device used to measure lactate was an electro-chemical based EKF Biosen C-line Sport (EKF Diagnostic, Magdeburg, Germany). The reported coefficient of variation (CV) for the equipment is 1.5% according the manufacturer. Blood gases were analyzed instantly on site using a GEM Premier 3000 (Instrumentation Laboratory, Lexington, MA, USA) that uses a potentiometric system for analysis. The manufacturer reports following precision: in pH 7.15 level standard deviation (SD) is 0.009 and in pH level 7.46 SD is 0.005. In addition, blood bicarbonate and base excess were calculated. The coefficient of variation for sodium and potassium measures was 0.86% and 0.71% in our laboratory, respectively. Hemoglobin concentrations was analysed using Sysmex KX 21 N (Kobe, Japan) with a CV < 1.5% in our laboratory.

### Nutrition

The participants were advised to maintain their normal dietary habits during the course of the study. Nutritional sports supplements (i.g. creatine, caffeine), except pure protein or carbohydrate, were forbidden during the study. All participants were instructed to keep a food diary 24 hours prior to each test. They were also instructed to eat as similarly (according to the first food diary) as possible before each test. The food diaries were analysed by using Micro Nutrica 3.0 software (Social Insurance Institution, Turku, Finland). The mean ± SD energy intake of four one day treatments was 3202 ± 478 kcal (carbohydrate 48 ± 4%, protein 24 ± 2%, and fat 28 ± 4%).

### Training

The participants were allowed to train normally according to their training program. All participants had a minimum of four years of competitive swimming experience. The study occurred in the beginning of their training season, so that every participant would be in the similar preparation phase. The swimmers had six training days and one rest day per week. The average amount of training sessions was nine, but some swimmers trained 11 times per week (Table 1). Average length of each training session was two hours. In addition to swimming, all participants participated in three resistance training sessions per week for 60 minutes per session.

**Table 1:** Average training week during the study

	**Days**						
**Variable**	**1**	**2**	**3**	**4**	**5**	**6**	**7**
**Physical activity**							
**Morning**	Aer	Aer/Anaer	Rest	Aer/Anaer	Drills	Strength	Rest
**Evening**	Speed	Strength	Speed	Strength	Speed	Aer/Anaer	
**Load**	Medium	High	Light	High	Light	High	

Aer = aerobic training, anaer = anaerobic training.

### Statistical methods

All results were analysed with SPSS-statistics program (PASW statistics 17). Means ± SDs were calculated and the Wilcoxon Signed Rank Test was used to evaluate the differences between the means. A nonparametric test was chosen because the data was not normally distributed tested with the Shapiro-Wilk test. Statistical comparisons were considered significant when p values were < 0.05.

## Results

Subjects reported no side effects related to SB intake, but symptoms of paraesthesia was experienced by all subjects consuming BA.

### Swimming times

**Figure 2:**
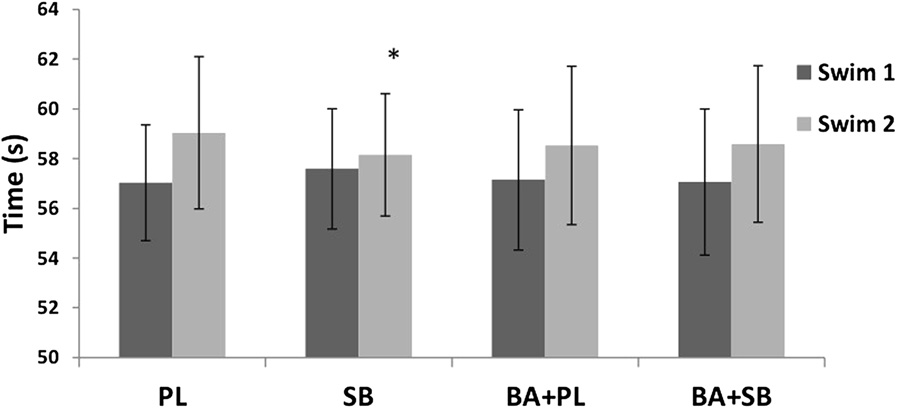
**Swimming times (mean ± SD) in the supplemented groups.** PL = placebo, SB = sodium bicarbonate, BA + PL = beta-alanine and placebo, BA + SB = beta-alanine and sodium bicarbonate, *Indicates a significant difference (p < 0.05) compared to PL.

There were no significant differences in the time of the first 100-m sprint between the groups. In the second 100-m swim, the increase in time of the second versus the first 100-m swimming time was 1.5 s less (p < 0.05) in the SB group compared to the PL group (Figure 2). No significant differences were noted between the first or second sprint in either BA + SB or BA + PL.

### Blood variables

#### Lactate, pH

**Figure 3:**
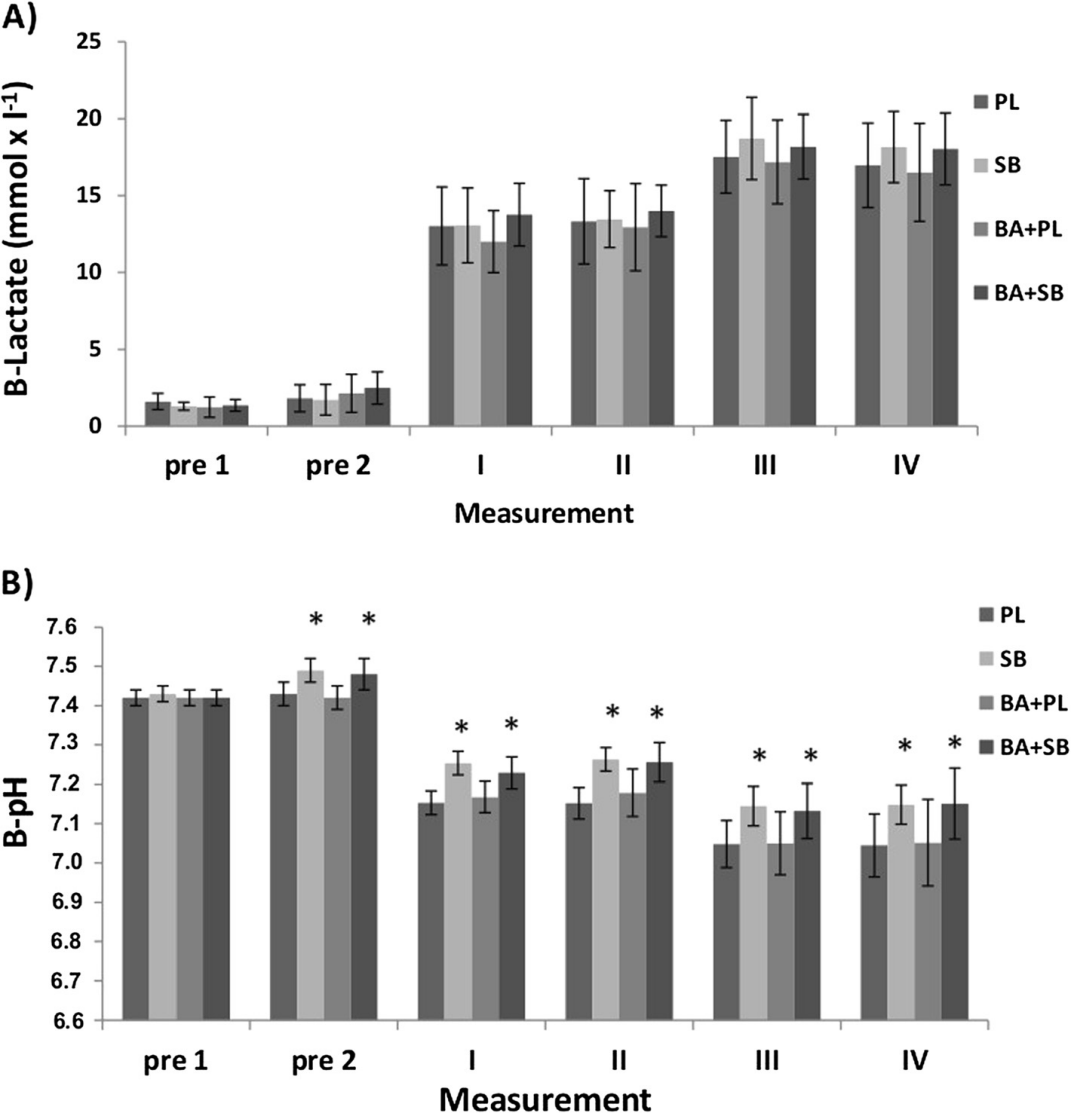
**Blood lactate and pH values (mean ± SD) in the supplemented groups in different measurement time points. A)** Blood lactate (B-Lactate), **B)** pH (B-pH), PL **=** placebo, SB **=** sodiumbicarbonate, BA + PL **=** beta-alanine and placebo, BA + SB **=** beta-alanine and sodium bicarbonate, pre 1 **=** 60 min before swimming, pre 2 **=** 2 min before swimming the first 100 m, I and III 2 min after both 100 m swimming, II and IV 8 min after both 100 m swimming, * Indicates a significant (p < 0.05) difference compared to PL.

There were no significant differences between the groups although lactates in measurements III and IV tended (p < 0.08-0.09) to be greater in SB supplemented groups (Figure 3A). Blood pH values (Figure 3B) were significantly (p < 0.05) greater in the SB and in the BA + SB combination group 2 min before the first swim and in all measurement points following swimming compared to the PL measurement values.

#### Sodium, potassium

**Figure 4:**
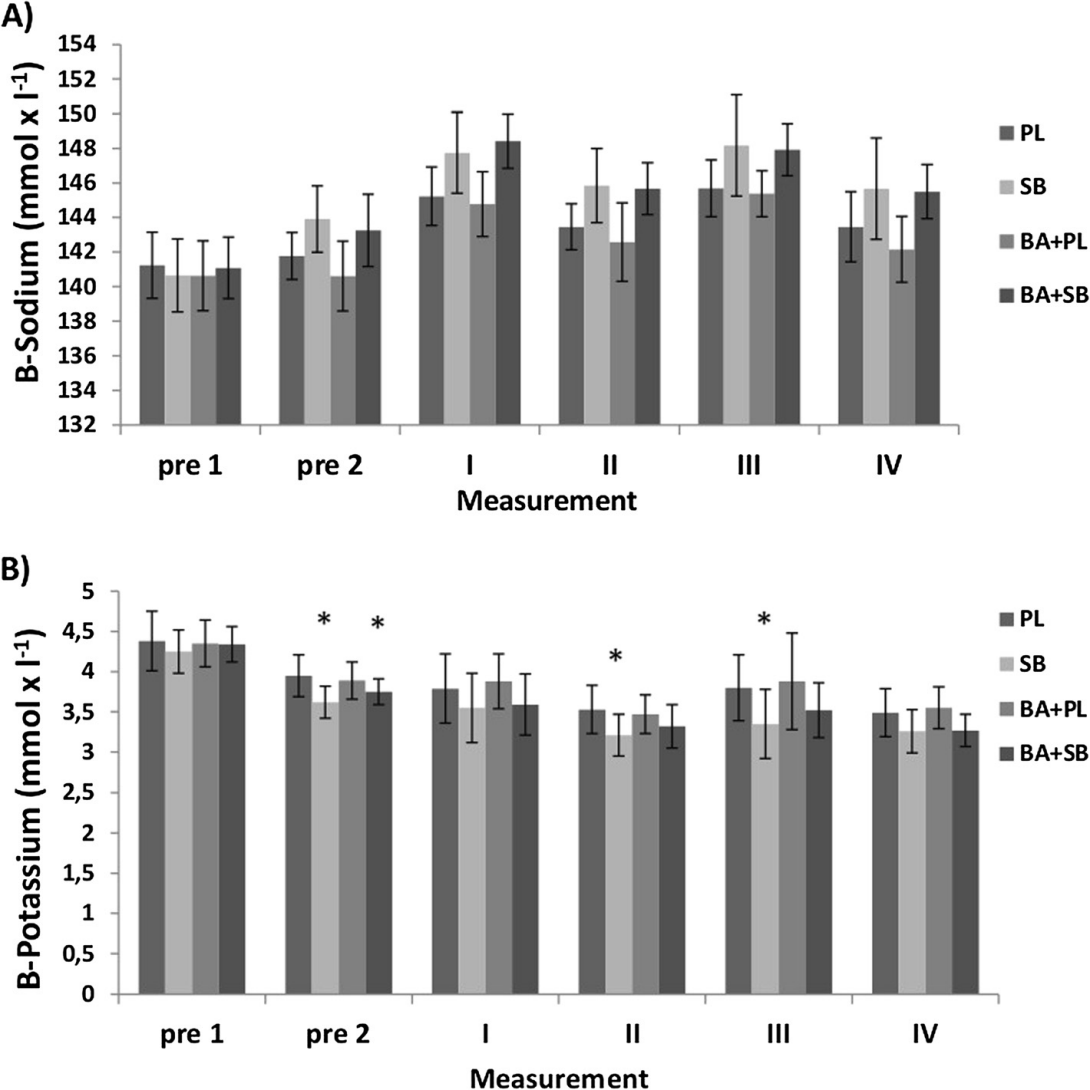
**Blood sodium and potassium values (mean ± SD) in the supplemented groups in different measurement time points. A)** Blood sodium (B-Sodium), **B)** potassium (B-Potassium), PL **=** placebo, SB **=** sodium bicarbonate, BA + PL **=** beta-alanine and placebo, BA + SB **=** beta-alanine and sodium bicarbonate**.** pre 1 = 60 min before swimming, pre 2 = 2 min before swimming the first 100 m, I and III 2 min after both 100 m swimming, II and IV 8 min after both 100 m swimming, * Indicates a significant (p < 0.05) difference compared to PL.

Significantly (p < 0.05) greater increases in plasma sodium concentrations were observed in SB and in BA + SB at every measurement point (except pre 1) compared to the PL values. A significant decrease in sodium concentrations was seen at BA + PL compared with PL during IV (Figure 4A). Significantly (p < 0.05) smaller plasma potassium concentrations were observed in SB and in the SB + BA groups at Pre 2, II and III compared to the PL values (Figure 4B).

### Bicarbonate, base excess

**Figure 5:**
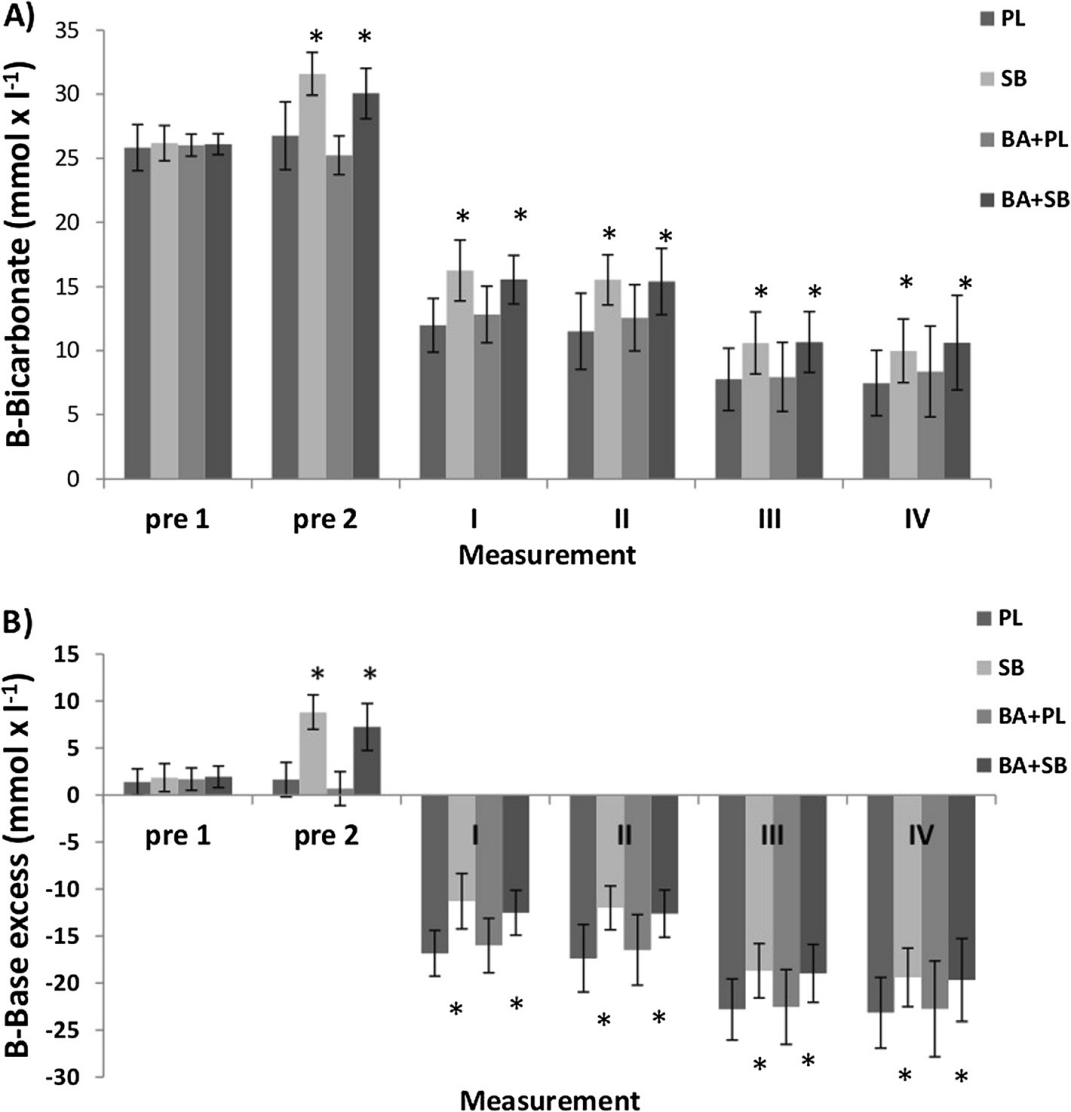
**Blood bicarbonate and base excess values (mean ± SD) in the supplemented groups in different measurement time points. A)** Blood bicarbonate (B-Bicarbonate) and **B)** base excess (B-Base excess) values (mean ± SD) in the supplemented groups in different measurement time points, PL = placebo, SB = sodium bicarbonate, BA + PL = beta-alanine and placebo, BA + SB = beta-alanine and sodium bicarbonate, pre 1 = 60 min before swimming, pre 2 = 2 min before swimming the first 100 m, I and III 2 min after both 100 m swimming, II and IV 8 min after both 100 m swimming, * Indicates a significant (p < 0.05) difference compared to PL.

There were significantly greater values in blood bicarbonate and base excess values in SB and in BA + SB at every measurement point (except pre 1) compared to the PL values (Figures 5A and 5B).

## Discussion

### Main results

The primary findings from this investigation were that there was a significantly less attenuation in swim time for the second 100 m sprint following SB supplementation. However, co-supplementation of SB with BA did not add any further benefit.

### Swim times

The subjects that ingested SB swam the second 100-m sprint 1.5 s (2.4%) faster compared to the PL trial. This improvement confirms the results previously reported by Mero et al. [23], who used a similar testing protocol and reported a 0.6 s (p < 0.05) improvement in subjects ingesting SB. Also in support of the present results, Gao et al. [4], demonstrated that pre - exercise supplementation with SB does not improve the first sprint but may be beneficial in later repetitions. In swimming races, an improvement of 1.5 s in performance may have a decisive effect on success in the final competition. During swimming competitions swimmers are often required to perform multiple heats during one day [32,33]. For example, in the 2012 London Olympics a female swimmer came in first in semifinals 200-m free style and then won the gold medal 15 min later in the 100-m backstroke. In our study the combined SB and BA supplementation did not improve swimming times. Recently Hobson et al. [26] showed that chronic BA and acute SB supplementation improved 2000 m rowing performance and they concluded that the addition of acute SB to chronic BA supplementation may further enhance rowing performance. They used a BA dose of 6.4 g per day during four weeks. In our study we used only 4.8 g per day for four weeks. Muscle carnosine is important in buffering and the muscle content is dependent on BA ingestion [15] it would be interesting to use either bigger doses and/or longer supplementation periods in future studies.

### Blood pH

Blood pH values were significantly greater when ingesting SB and with SB plus BA supplementation prior to the first swim compared to PL. The increase in blood pH was similar as in the earlier studies because the SB dose (0.3g·kg^-1^ body mass) used was comparable. However, time for the first swim trial was not improved with SB or with SB + BA ingestion. In all four treatments following the swim trials, blood pH values were significantly lower compared to pre-values. Consequently, the second swim trial was performed in stronger acidosis than the first, and in this state the best performances were seen during SB treatment. These results in part confirm those by Gordon et al. [34], who observed that the alkalotic condition attenuates the increase in blood H^+^ concentration. We hypothesized that the extracellular buffering action of SB and the intracellular pH-buffering action of carnosine through BA ingestion would be additive, resulting in an increased protection against the acidosis produced during anaerobic interval swimming. Our results appear to support the work of Hobson et al. [20] that suggested that benefits of BA supplementation may be dependent upon high intensity exercise durations lasting more than 60 s. However, it was a bit surprising that when SB and BA were combined the benefit observed with SB only was negated. This is difficult to explain but, although speculative, it may be related to muscle carnosine concentations. Although several studies have suggested that trained anaerobic athletes have higher muscle carnosine concentrations [35-37], the ability to enhance muscle carnosine concentration from training only has not been established. Therefore, the effect of supplementing for some individuals may be small. It is possible that the effect of lowering intracellular acidity in this type of exercise is not the only factor for muscle fatigue [38]. The other possible factors for muscle fatigue may be phosphocreatine stores, maximal oxygen uptake and some neural factors.

### Blood lactate

There were no significant differences in blood lactate concentrations between the treatment groups, although it seems to be higher with SB and SB + BA supplementation indicating increased buffering activity in muscle. The increase in peak blood lactate (change between PL and the SB groups) was about 1 mmol·l^-1^. This change was smaller than reported by Ibanez et al. [39] who demonstrated a difference in peak blood lactate between treatments of 2 mmol·l^-1^or more is needed to observe a strong and significant improvement in performance following SB supplementation. During intensive anaerobic work [40,41], it has been shown that lactate produced in fast-twitch muscle fibers can circulate to other fast-twitch or slow-twitch fibers for conversion to pyruvate. Pyruvate, in turn, converts to acetyl-CoA for entry into the citric acid cycle for aerobic energy metabolism. Lactate shuttling between cells enables glycogenolysis in one cell to supply other cells with fuel for oxidation [42]. Swimmers generally utilize both aerobic and combined aerobic-anaerobic training, and this may enhance the capacity of the lactate shuttling. In the present study, the effect of this lactate shuttling on peak blood lactate values remains an open question. However, the performance in the second 100 m swim following SB supplementation improved compared to PL, but co-supplementation with SB and BA did not confer any further significant benefit.

### Blood bicarbonate, base excess, sodium and potassium

Blood analyses confirmed that SB and SB with BA were successful in increasing blood bicarbonate concentration and base excess consistent with previous studies of SB ingestion [43]. Both SB and SB with BA supplementations increased blood sodium as expected but blood potassium decreased. Earlier Sostaric et al. [30] reported that SB supplementation lowered circulating potassium and enhanced muscle potassium uptake, sodium delivery and chloride uptake with alkalosis. These physiological changes are all consistent with preservation of membrane excitability during exercise [30]. This suggests that lesser exercise-induced membrane depolarization may be an important mechanism underlying enhanced exercise performance with alkalosis. Thus alkalosis is associated with improved regulation of potassium, sodium, chloride and lactate. On the other hand, ingestion of BA significantly decreased blood sodium 8 min after the second swim compared with placebo, and tended to decrease at the other measurement points. Whereas potassium levels were very similar compared to placebo treatment. Consequently, ingestion of BA may affect membrane excitability differently during exercise compared with SB ingestion.

## Conclusions

The results of this study indicate that there was a significant improvement in swimming time during the second 100 m swim trial following acute SB supplementation compared to PL, but the addition of chronic BA to acute SB did not provide any additional ergogenic benefit. Results indicate the efficacy of SB supplementation when performing maximal interval swimming lasting under 60 s but do not support any additional benefit of SB combined with BA.

## Competing interests

The authors declare that they have no competing interests.

## Authors’ contributions

AAM (corresponding author) was responsible for the study design, the execution of the measurements, the statistical analysis and the writing of the manuscript. PH and JS participated in the study design, execution of the measurements, the statistical analysis and the writing of the manuscript. JRH and JRS participated in the study design and in the writing of the manuscript. All authors read and approved the final manuscript.
